# The retinal pigment epithelium undergoes massive apoptosis during early differentiation and pigmentation of the optic cup

**Published:** 2011-04-20

**Authors:** M.O. Pequignot, A.C. Provost, S. Sallé, M. Menasche, S. Saule, J-P. Jaïs, M. Abitbol

**Affiliations:** 1 UNIVERSITE PARIS DESCARTES, CERTO, EA # MRES 2502, Faculté de Médecine PARIS DESCARTES - site Necker, Paris, France and AP-HP, Department of Ophthalmology, CHU NECKER-ENFANTS MALADES, Paris, France; 2 CNRS, UMR 146, Institut Curie, Orsay, France; 3 Service de Biostatistiques et d’Informatique médicale, Hôpital Necker, Paris, France; 4 Inserm U1051, Institute for Neurosciences of Montpellier, Montpellier, France

## Abstract

**Purpose:**

The aim of our work was to study apoptosis during the development of the retinal pigment epithelium (RPE) in mice between embryonic day (E) 10.5 and E12.5 and to examine a possible link between apoptosis and pigmentation.

**Methods:**

We collected mouse embryos at E10.5, E11.5, and E12.5 and labeled apoptotic cells in 5-µm paraffin sections, using the terminal deoxynucleotidyl transferase dUTP nick end labeling technique. We counted the total number of cells and the number of apoptotic cells in the early developing RPE and calculated the percentage of apoptosis at each stage.

**Results:**

In the C57BL/6J mouse, 17% of the RPE cells were apoptotic at E10.5 compared to 0.9% at E12.5. At E11.5, three-quarters of the RPE cells began to pigment, and apoptotic cells were located mostly in the nonpigmented part. In contrast, in the BALB/c mouse (tyrosinase-deficient) and pJ mouse (carrying mutations in the *p* gene) hypopigmented strains, the RPE contained significantly fewer apoptotic cells (7.5% and 10.1%, respectively) at E10.5 than controls. Subsequently at E11.5 and E12.5, the two hypopigmented strains displayed different apoptotic patterns; the BALB/c RPE had a similar percentage of apoptotic cells to controls (1.5% and 1.1%, respectively, for BALB/c versus 3.0% and 0.9%, respectively, for C57BL/6J), whereas the pJ RPE contained significantly more apoptosis (7.5% and 3.5%, respectively). Overall we observed differences in the evolution of the relative total number of RPE cells between the three strains.

**Conclusions:**

Apoptosis is a main event during the first stages of normal RPE development, indicating an essential role during RPE differentiation. Moreover, the early apoptotic pattern and possibly the whole early development of the RPE is different between hypopigmented and pigmented strains, as well as between BALB/c and pJ mice. This suggests the existence of regulatory and developmental differences with a more complex origin than just differing pigmentation levels.

## Introduction

In eukaryotes, apoptosis occurs in balance with mitosis in adults and during development. The development of organs combines waves of cell divisions and differentiating processes as well as the disappearance of transitory structures and the elimination of abnormally differentiated cells. Apoptosis is particularly important in the central nervous system, where a lack of this programmed form of cell death [1-3] is detrimental. In the retina as in other tissues, apoptosis plays an important role [4,5] at different stages of development.

At the beginning of retinal development, the optic vesicle develops as a diverticulum arising laterally from the neural tube and converting into an optic cup, composed of an outer and an inner layer. Despite their identical embryonic origin, these layers differentiate into two distinct tissues. The inner layer of the optic cup differentiates during the pre- and post-natal period into a multilayered structure [6], termed the neuroretina, containing the photoreceptors, the ganglion cells, and other retinal neurons. The outer layer forms the retinal pigment epithelium (RPE, or pigmented retina), a single layer of non-neuronal, pigmented, cuboidal cells. The RPE [7] has phagocytic capacities and is necessary for proper neuroretinal functioning.

The neuroretina and the RPE interact with each other during development, and their interaction is crucial for the proper differentiation of both tissues. In particular, it has long been shown in many species that an anomaly in RPE pigmentation, such as albinism, leads to multiple abnormalities in neuroretinal development. These comprise an abnormal apoptosis and mitosis pattern during post-natal development [7-9], an abnormal division pattern of the retinal progenitors from the early stages [10-13], an abnormal decussation of the ganglion cell axons [9,13], and a decrease in photoreceptor numbers in adulthood [10,13,14]. Although the early albino neuroretinal development has been studied extensively for many years, studies on the early development of the albino RPE itself are rare. Ilia and Jeffery [15] compared mitotic and pyknotic profiles in the rat from embryonic day (E) 12.5 to post-natal day (P) 11; Trousse et al. [16] qualitatively described apoptosis in the first stages of albino optic cup development in chicken and mouse and Laemle et al. [17] in a hypopigmented mouse. However, there is no available data for pigmented strains of these species.

Previously we demonstrated that in the pigmented C57BL/6J mouse, the future neuroretina undergoes apoptosis during E10.5, the first stage of the mouse optic cup development, with 1.9% of apoptotic cells. This apoptotic cell level [18] decreases to 0.95% at E11.5 and 0.65% at E12.5 and to less than 0.2% thereafter. During this previous study, we also observed multiple apoptotic cells in the forming RPE. Here, we quantify this apoptosis in the RPE during the early developmental window E10.5 to E12.5 in the pigmented C57BL/6J strain. As RPE cellular pigmentation occurs during the same developmental period, we quantified apoptosis in parallel in two hypopigmented strains (BALB/c and pJ). We showed that the apoptosis pattern in RPE is different between normally and hypopigmented strains as well as between the two hypopigmented strains.

## Methods

### Animals

All animals were handled in accordance with the ARVO Statement for the Use of Animals in Ophthalmic Research and all experiments were approved by our institution’s animal care board. The pigmented C57BL/6J (B6) mouse strain (Janvier, Le Genest, France) was chosen as a reference. The albino BALB/c Nj mouse strain (Janvier) carries a point mutation in the tyrosinase gene, at nucleotide residue 387 (G-to-C transition), causing a Cys→Ser substitution at position 85 in one of the cysteine-rich domains of the molecule. The mutated protein [19,20] is not functional. Although a tyrosinase-knockout mouse exists on a B6 genetic background (Jackson Laboratory, strain B6(Cg)-Tyr^c-2J^/J), we did not use this strain as it shows abnormal photoreceptor apoptosis during young adulthood, which is not present in the pigmented B6 strain [21] or in the BALB/c strain. The hypopigmented pJ strain carries a spontaneous 350-bp deletion in the *p* gene that plays a role in melanogenesis. The deletion results in a frameshift [22], which generates a protein truncated at amino acid position 689 and which is not functional. The pJ strain has a B6 genetic background and was kindly provided by [23] Murray Brilliant. The fasl*^gld^* strain (on a B6 background, Jackson Laboratory, Bar Harbor, ME) carries a T→C transition point mutation near the 3′ end of the Fasligand (*Fasl*) coding sequence [24] that replaces a highly conserved phenylalanine with a leucine at position 273 in the extracellular region of the encoded protein. This protein shows a weak [25], if any, interaction with Fas.

### Histology

Embryos were obtained in our facility and numbered with day 0.5 taken as the morning of vaginal plug observation. Pregnant females were killed by lethal chloral hydrate intra-peritoneal injection at the indicated gestation times. Embryos were removed and fixed in 4% PFA at 4 °C for at least 36 h [18]. They were then embedded in paraffin, and cut into 5-μm sagittal sections. In addition, some of the B6 embryos were cut into 5-μm frontal sections.

### TdT dUTP nick end labeling and counting

For each developmental stage, we used at least four animals (from at least two different litters). The number of embryos used for each stage and genotype is indicated in Table 1, as is the mean number of sections analyzed for each embryo. The TdT dUTP Nick End Labeling (TUNEL) assay [18] was performed with the DeadEnd Colorimetric kit (Promega, Charbonnières-les-Bains, France), according to the manufacturer’s protocol. All slides were analyzed by the same investigator (1,000× magnification). Another investigator independently counted apoptotic cells on 10% of the sections, randomly chosen, and obtained similar results (less than 5% variation). Sections to be analyzed were chosen around the maximal diameter and were not adjacent to avoid counting the same apoptotic cell twice. For each section we counted the total number of RPE cells and the number of apoptotic RPE cells and calculated the percentage of apoptosis=[apoptotic RPE cell number/total RPE cell number] × 100. At E11.5 and E12.5, the RPE and the ciliary margin zone were clearly differentiated, as the RPE is monolayered and the two cell types are morphologically different. At E10.5, this difference was less evident, so we considered the transition between these two parts to be at the plane perpendicular to the tip of the subretinal space.

**Table 1:** Number of embryos and mean number of sections analyzed per embryo.

**Stage**	**C57BL/6J**		**BALB/c**		**pJ**	
	**Embryos**	**Sections**	**Embryos**	**Sections**	**Embryos**	**Sections**
E10.5	13	9	5	6	4	7
E11.5	8	9	4	9	5	6
E12.5	5	8	4	10	4	11

Embryos from at least two different litters were analyzed per stage and per genotype.

### Statistics

The apoptotic cell counts were analyzed [26] by a generalized linear model with a Poisson distribution and a logarithmic link. The log of the total cell count was introduced in the model as an offset. To take into account the clustering of the data by animal and slide, we used the generalized estimating equation approach [27] to obtain nonbiased variances of the estimates and 95% confidence intervals of the cellular counts. Groups were compared by building and testing the corresponding contrast on the model parameters. The GENMOD function of the SAS 8.2 (SAS Institute Inc., Cary, NC) package was used for these analyses. The Bonferroni method was applied for multiple comparisons.

## Results

### Pigmentation occurs at embryonic day 11.5

**Figure 1:**
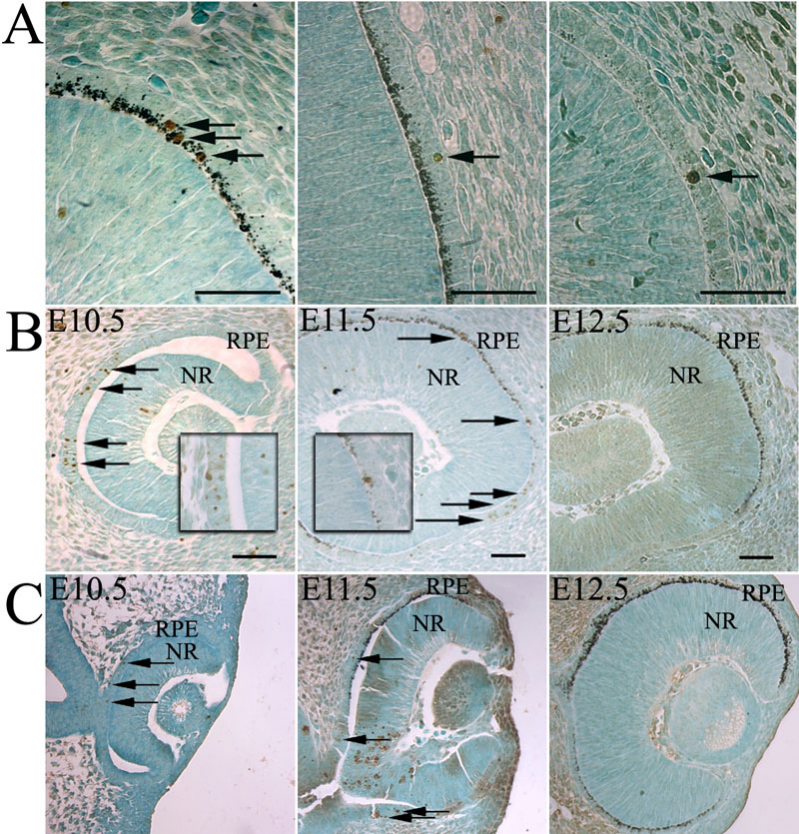
Apoptosis is massive in the B6 retinal pigment epithelium at E10.5. **A**: On sagittal sections of embryonic day (E) 12.5 (left and middle panel) and E11.5 (right panel), B6 RPE is shown at the same magnification used for counting. We can easily differentiate melanosomes (small black dots) and terminal deoxynucleotidyl transferase dUTP nick end labeling (TUNEL)-positive cells (brown-labeled dots, indicated by arrows). **B**: Micrographs in **B** show sagittal sections of E10.5, E11.5, and E12.5 B6 optic cups after a TUNEL assay. The insets in E10.5 and E11.5 show higher magnification of apoptotic cells at the corresponding stages. Panel **C** shows micrographs of frontal sections of E10.5, E11.5, and E12.5 B6 optic cups after a TUNEL assay. RPE, retinal pigment epithelium; NR, neuroretina; Scale bars represent 100 μm. Arrows indicate examples of RPE-labeled cells (apoptotic cells can also be observed in the neuroretina), which are predominantly located in the ventral nonpigmented part.

We chose as a reference the pigmented B6 strain, considered to have a standard optic development. It was first possible to count cells at E10.5, when the optic cup had just formed. At this stage in B6 mice, we observed that the RPE is not pigmented. The most peripheral RPE, at the junction with the neuroretina, still consisted of multiple cell layers, while the central part was monolayered. Pigmentation first appeared at E11.5, extending from the dorsal pole and covering approximately 75% (in sagittal sections) of the RPE layer. In the frontal sections we confirmed that the pigmentation was present in the dorsal pole and extended to the optic stalk, although it was less obvious. At E12.5, almost all cells constituted a monolayer and were pigmented. The melanosomes, as shown in Figure 1A appeared as small black dots in the basal third of the cells and were easily distinguishable from the TUNEL labeling (brown).

### High proportion of apoptotic cells in B6 optic cups at embryonic day 10.5

We first studied sagittal sections of optic cups. At E10.5, 17.3% of the cells in the RPE, as indicated in Table 2, were TUNEL labeled. These cells, as visible in Figure 1B, were mostly localized dorsally near the optic stalk. At E11.5, only 3% of all RPE cells were labeled, and these cells were mostly at the ventral pole of the optic cup, at the junction with the neuroretina where RPE cells were not yet pigmented. Finally, at E12.5 only a small percentage of RPE cells were labeled (0.9%). Pairwise comparisons, clearly summarized in Table 2, showed significant differences (p<0.0001) in the percentage of labeled cells at all three stages. To localize precisely the apoptotic cells in three dimensions, we analyzed frontal sections of embryos at each stage with the TUNEL technique. Similarly to what we observed in sagittal sections, we found apoptotic cells near the optic stalk, as shown in Figure 1C, at stage E10.5. At E11.5, pigmented cells covered the internal half of the RPE. The most external cells of the RPE were not yet pigmented. Apoptotic cells were mostly found [28], as described previously, in the nonpigmented half of the layer and at the borders of the closing embryonic fissure. At E12.5, we found only a small number of TUNEL-positive cells. Thus, the three-dimensional dying cell wave [6] begins at the optic stalk and moves forward within the optic cup from the central to the peripheral RPE, as reported before for the time of neuroretinal cell birth. We counted the number of apoptotic cells in the pigmented and nonpigmented regions at E11.5, an intermediate stage for the pigmentation, in sagittal sections. Apoptotic cells in the nonpigmented region made up 14.1%, a similar percentage to that observed at E10.5 (i.e., 17.3%), and 1.8% of cells in the pigmented part of the RPE were apoptotic, which is closer to the percentage found at E12.5 (i.e., 0.9%). The apoptotic pattern seemed to peak just before the time when RPE cells became pigmented.

**Table 2:** Percentage of apoptotic cells in B6, BALB/c and pJ RPE at E10.5 to E12.5.

**Stage**	**C57BL/6J**	**BALB/c**	**pJ**
E10.5	17.3 [15.9–18.8]	7.5 [5.1–11.0] ***	10.1 [9.1–12.2] ****
E11.5	3.0 [1.9–4.8]	1.5 [1.0–2.3] ns	7.4 [5.4–10.3] §
E12.5	0.9 [0.6–1.2]	1.1 [0.7–1.6] ns	3.5 [2.9–4.3] ****

Confidence intervals are indicated in brackets. p<0.0001 between all B6 stages. § p=0.015, *** p<0.001, **** p<0.0001 between hypopigmented strains and B6 for a given stage. ns: non significant.

### The apoptotic pattern is different in hypopigmented and nonpigmented strains

**Figure 2:**
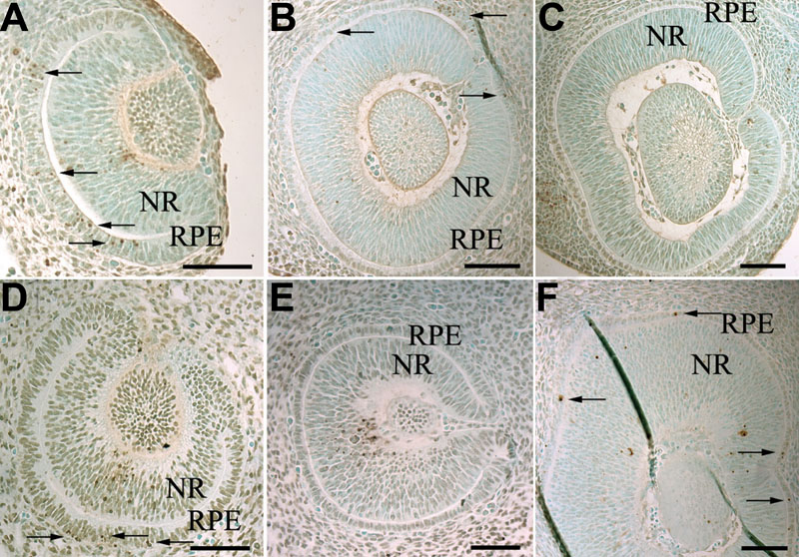
The percentage of apoptotic cells is lower in the two strains with hypopigmented  retinal pigment epithelium. The micrographs of the upper pannel show sagittal sections of embryonic (E) day 10.5 (**A**), E11.5 (**B**), and E12.5 (**C**) BALB/c optic cups and those of the lower panel show sagittal sections of E10.5 (**D**), E11.5 (**E**), E12.5 (**F**) pJ optic cups after a terminal deoxynucleotidyl transferase dUTP nick end labeling (TUNEL) assay. RPE, retinal pigment epithelium; NR, neuroretina. Scale bars represent 100 micronmeters. Arrows indicate labeled cells.

We then studied apoptosis in two mouse strains with hypopigmented eyes: the BALB/c and the pJ strains. BALB/c mice carry a mutation in the gene encoding tyrosinase (*Tyr*), the enzyme required for the key step of melanogenesis. These mice [20] are white with red eyes. pJ mice carry a mutation in the *p* gene. When this enzyme is lacking, the pH inside melanosomes is abnormal. The pJ mice [23] are pearl gray with red eyes. The development of the optic cup in the two types of embryo appeared structurally normal. We dissected embryos from these two strains at the same stages as B6 mice and analyzed the sections with the TUNEL technique. Table 2 shows unequivocally that at E10.5 there was a significant difference in the percentage of dying cells between B6 (17.3%) and the nonpigmented strains (7.5 and 10.1%). In contrast, at E11.5 and E12.5 we observed a similar percentage of apoptotic cells in B6 (3%) and BALB/c (1.5%) strains, whereas the pJ strain showed a higher percentage (7.4%) of apoptotic cells. This difference was associated with a change in the location of the dying cells; at E11.5, the apoptotic cells were mostly near the external pole in B6 and BALB/c, whereas in pJ embryos they were still near the optic cup, at the internal pole. This is reminiscent of the situation observed in B6 optic cups at E10.5, as Figure 2 illustrates it unambiguously. At E12.5 we found TUNEL-labeled cells laterally in pJ optic cups. The differences between B6 and nonpigmented strains at E10.5 suggested that the development might be delayed in hypopigmented RPE.

### The retinal pigment epithelium cell number evolves differently between strains

We counted the total number of RPE cells on all sections of the three strains at the three stages and compared the evolution between the strains. The results displayed in Table 3 lead to the surprising conclusion that the three strains showed a different progression in number over time. In B6 embryos, the number of RPE cells progressed continuously from a mean of 136.2 cells/section at E10.5 to reach 178.3 cells/section at E11.5 and 221.5 cells/section at E12.5. In the BALB/c animals, the total number of RPE cells was higher at E10.5, with 170.7 cells/section, but grew more slowly with 188.3 cells/section at E11.5 and 210.7 cells/section at E12.5. Finally, in pJ embryos, the number of RPE cells was much higher at E10.5, with 228.9 cells/section, and then decreased to 141.8 cells/section at E11.5 and 180.2 cells/section at E12.5.

**Table 3:** Total number of RPE cells/section in B6, BALB/c and pJ RPE at E10.5 to E12.5.

**Stage**	**C57BL/6J ^(1)^**	**BALB/c ^(2)^**	**pJ ^(3)^**
E10.5	136.2 [131.4–141.2]	170.7 [175.6–165.4] ns	228,9 [223.5–234.4] **** §§§
E11.5	178.3 [173.2–183.6]	188.3 [183.1–193.7] ns	141.8 [137.0–146.9] * §
E12.5	221.5 [216.2–227.0]	210.7 [205.4–216.1] ns	180.2 [175.2–185.5] ** §

Confidence intervals are indicated in brackets. ns: non significant. * and § p<0.05; ** p<0.01; *** and §§§ p<0.001; **** p<0.0001. * indicates p values between pJ and B6. § indicates p values between pJ and BALB/c. ^(1)^ p<0.001 between all B6 stages. ^(2)^ p=0.057 between E12.5 and E10.5 in BALB/c. The total RPE cell numbers between the other stages are not statistically different. ^(3)^ p<0.0001 between E11.5 and E10.5; p=0.0011 between E12.5 and E10.5; p<0.05 between E12.5 and E11.5.

### The FAS apoptotic pathway is involved in the apoptosis observed at embryonic day 10.5

We assayed whether the FAS apoptotic pathway was implicated in the high percentage of cell death observed in B6 optic cups at E10.5. For this purpose we measured the occurrence of cell death in the fas*^gld^* embryo RPE. This strain is mutated [24,29] in the *Fasl* gene, leading to the production of a protein unable to bind its receptor. In the fas*^gld^* RPE, the total number of cells is similar to that of the B6 RPE. In contrast, 7.5% (5.8%–9.7%) of cells were apoptotic versus 17.3% (15.9%–18.8%) in the B6 RPE (p<0.0001). Thus, the absence of a functional Fasl protein resulted in the immediate survival of more than 50% of the cells as compared to control RPE.

## Discussion

We previously showed the occurrence of apoptosis in the future B6 neuroretina in the pigmented mouse at E10.5 [18], just at the stage when the optic vesicle is formed. Here, we show that this phenomenon is not restricted to the neuroretina but also involves the RPE at the same time. The developing RPE is more dramatically affected than the developing neuroretina at this stage, as 17% of the cells were apoptotic at this stage (versus 2% in the neuroretina).

The apoptotic wave occurring in the developing RPE follows the developmental wave, beginning dorsally at the optic stalk and moving forward to its distal cells. This is a dramatic and rapid process, which suggests that this phenomenon is an important step in eye development. The apoptotic wave is not detected in the pigmented rat at E12.5 (equivalent to E10.5 in mouse) [15]. However, the correspondence between the stages in different species is not precise, and as the peak of apoptosis is a rapid event, it may occur earlier than E12.5. Moreover, many apoptotic cells have also been observed in albino chicken from similar stages [16] (stage Hamburger–Hamilton 17, equivalent to E10.5 in mouse). Therefore, it would be important to quantify apoptosis in pigmented rat and chicken but also other species systematically during the first stages of optic cup construction to verify if this peak of apoptosis is an evolutionary-conserved phenomenon.

We show here an involvement of the Fas pathway in RPE apoptosis at E10.5 in the mouse, with about 50% less apoptotic RPE cells observed in fas*^gld^* embryos than in controls. This twofold decrease in the apoptotic ratio [18] is similar to the one observed in the neuroretina of fas*^gld^* embryos at the same stage. In the neuroretina the cells that survived this period of apoptosis must die subsequently as the adult fas*^gld^* retina does not contain cells in excess as compared to controls. It is possible that the apoptosis is also only delayed in fas*^gld^* RPE. As in the neuroretina, cell death is not abolished in the absence of the *Fasl* pathways in the RPE, most likely because other apoptotic pathways, such as the Bax/Bcl2 pathway, are also involved.

We observed a different pattern of apoptosis and different total numbers of RPE cells between the pigmented B6 strain and the hypopigmented BALB/c and pJ strains. An abnormal development of the neuroretina and RPE has already been described in all hypopigmented strains. This has been attributed to the effect of the strong reduction of 3,4-dihydroxyphénylalanine (L-DOPA) [15,30], a melanin precursor that is the product of the tyrosinase enzyme, which catalyzes the first and key steps of melanization. L-DOPA acts on cell-cycle exit of the neuroretinal neuroblasts during development. In post-natal rat neuroretinas, it has been demonstrated that in the absence of tyrosinase (in tyr- albino animals), a prolonged state of developmental immaturity is observed [15,30], the number of mitotic cells increases, and the number of apoptotic cells decreases, inducing a transitory increase of cells in the neuroblast layer. Ilia and Jeffery [8,15] studied RPE mitosis in rat embryos from E12.5 (pigmentation occurs at E13.5 in rat and at E11.5 in mouse). They showed that in tyrosinase-deficient rats, RPE development is also disturbed; more mitotic cells are observed and the developmental wave of mitosis from center to periphery is abolished. Similar results have been published for mitosis in *p*-deficient mice. We also observed these developmental wave anomalies, at the stages studied, in pJ strains but not in the BALB/c mice.

Beerman et al. [31] showed in the noncongenic albino Naval Medical Research Institute (NMRI) strain that tyrosinase is not expressed at E9.5 but is expressed at E10.5 in the whole presumptive RPE. We could assume that in pigmented strains, L-DOPA is actively produced [13] as early as E10.5, whereas tyrosinase-deficient strains produce only trace levels. In tyrosinase^+^ hypopigmented strains, tyrosinase is produced but mostly either mislocalized or misfolded. The adult mice are slightly pigmented, so the L-DOPA levels could be intermediate at the first stages. This intermediate level of L-DOPA could explain the intermediate apoptotic rate observed in the pJ strain compared to those of BALB/c and B6 strains at E10.5.

At E10.5, the total number of cells between the three strains is different and continues to evolve differently thereafter. The reduction of L-DOPA should provoke an increased mitosis and reduced apoptosis, thus resulting in an increased number of RPE cells. Consistently, we observed in both hypopigmented strains, although more pronounced in pJ animals, an increased number of total RPE cells at E10.5 compared to the pigmented control. However, surprisingly in BALB/c mice this number increased more slowly at subsequent stages than in controls, and RPE cell number in pJ mice clearly decreased. These results suggest that there is another factor affecting early RPE differentiation acting in parallel to the formation of L-DOPA.

In conclusion, our data propose that the albinism phenotype begins with RPE anomalies at the first stages of optic cup differentiation and differing L-DOPA levels are not the sole underlying cause. Moreover, this study lays the groundwork for future investigations aimed at unraveling the molecular mechanisms underlying the developmental apoptosis affecting the RPE cell layer.
